# iTRAQ proteomics reveals the regulatory response to *Magnaporthe oryzae* in durable resistant vs. susceptible rice genotypes

**DOI:** 10.1371/journal.pone.0227470

**Published:** 2020-01-10

**Authors:** Zuobin Ma, Lili Wang, Mingzhu Zhao, Shuang Gu, Changhua Wang, Jiaming Zhao, Zhiqiang Tang, Hong Gao, Liying Zhang, Liang Fu, Yongan Yin, Na He, Wenjing Zheng, Zhengjin Xu

**Affiliations:** 1 Rice Research Institute of Shenyang Agriculture University, Shenyang, China; 2 Rice Research Institute of Liaoning Province, Liaoning Academy of Agricultural Sciences, Shenyang, China; 3 Sorghum Research Institute of Liaoning Province, Liaoning Academy of Agricultural Sciences, Shenyang, China; 4 Xinjiang Institute of Ecology and Geography, Chinese Academy of Sciences, Urumqi, Xinjiang, China; National Institute of Technology Rourkela, INDIA

## Abstract

Rice blast disease caused by *Magnaporthe oryzae (M*. *oryzae)* is one of the most serious diseases. Although previous research using two-dimensional gel-based proteomics to assess the proteins related to the rice blast resistance *had been done*, few proteins were identified. Here, we used the iTRAQ method to detect the differentially expressed proteins (DEPs) in the durable resistant rice variety Gangyuan8 (GY8) and the susceptible rice variety Lijiangxintuanheigu (LTH) in response to *M*. *oryza*e invasion, and then transcriptome sequencing was used to assist analysis A total of 193 and 672 DEPs were specifically identified in GY8 and LTH, respectively, with only 46 similarly expressed DEPs being shared by GY8 and LTH.39 DEPs involved in plant-pathogen interaction, plant hormone signal transduction, fatty acid metabolism and peroxisome biosynthesis were significantly different between compatible interaction (LTH) and incompatible interaction (GY8). Some proteins participated in peroxide signal transduction and biosynthesis was down-regulated in GY8 but up-regulated in LTH. A lot of genes encoding pathogenesis-related gene (PR), such as chitinase and glucanase, were significantly up-regulated at both the transcriptome and proteome levels at 24 hours post-inoculation in GY8, but up-regulated at the transcriptome level and down-regulated at the proteome level in LTH. Our study reveals that the pathogen-associated molecular pattern (PAMP)-triggered immunity defense system may be activated at the transcriptome level but was inhibited at the protein level in susceptible rice varieties after inoculation. The results may facilitate future studies of the molecular mechanisms of rice blast resistance.

## Introduction

Rice blast, caused by *Magnaporthe oryzae* L., is the most destructive disease in rice (*Oryza sativa*) [1] and reduces global yields annually by 10%–15% [2, 3].Knowledge on the broad-spectrum resistance mechanism to rice blast is of great significance for the disease resistance breeding and diseases control.

To combat *M*. *oryzae*, rice has formed two immune system, named pathogen-associated molecular pattern (PAMP)-triggered immunity (PTI) and effector-triggered immunity (ETI) [4, 5]. PTI was considered the first line of defense and conferd durable and broad-spectrum resistance. In this system, pattern recognition receptors (PRRs) can recognize PAMPs which are widely conserved in pathogens trigger a weakly immune response [6]. Plant PRRs contained transmembrane receptor-like kinases (RLKs) and receptor-like proteins (RLPs). Previous identified PAMPs included flagellin, peptidoglycan, lipopolysaccharide, and chitin [7–11]. ETI, as the second layer of the plant innate immune, is initiated by archetypical resistance (R) proteins that directly or indirectly recognize effectors secreted into the plant cells by the pathogen. Unlike PAMPs, effectors are highly variable, and the disease resistance mediated by ETI is generally race-specific [6].

Although PTI and ETI use different receptors, they share similar molecular processes, and both ETI and PTI can activate the expression of downstream response proteins. Between compatible and incompatible interactions, the expression profile of response proteins differs significantly [12–17]. Proteomic studies on the expression patterns and regulatory networks of these proteins are important for understanding the mechanisms of plant resistance.

Previous proteomics-based profiling studies on the rice response to rice blast fungus and elicitors have been performed using two-dimensional gel (2-DE) [18–20]. These studies identified diverse proteins responsive to *M*. *oryzae*, and some elicitors, such as reactive oxygen species (ROS) responses and the cellular protection of ROS production, NADPH-dependent thioredoxin reductase B lactoylglutathione lyase and uridine ribohydrolase 1 [18], receptor-like protein kinases (RLK), glucanases, peroxidase (POX 22.3), probenazole-inducible protein (PBZ1), and rice pathogenesis-related proteins (PR), were induced or increased in the inoculated leaf [20]. Although 2-DE is able to separate thousands of different proteins and provide visual information relating to the proteome, including under blast fungus invasion, it is not suitable for the detection of low-abundance proteins or high-accuracy quantification [21]. Therefore, many low-abundance differentially expressed proteins (DEPs) associated with blast resistance remain to be explored.

iTRAQ is a mass spectrometry-based quantitative approach that has become prevalent in rice proteomics [22–24]. In this study, we used iTRAQ technology to compare the protein expression profiles of compatible and incompatible interactions at 24 hours post-inoculation (hpi) by *M*. *oryzae* and used RNA-sequencing to analysis transcriptome differences. Our study revealed that several pathways, including plant–pathogen interaction, plant hormone signal transduction, fatty acid metabolism, and peroxisome biosynthesis, were specifically restrained in the susceptible variety.WRKY, C2H2, and other transcription factors (TFs) were detected in the transcriptome than in the proteome, and Plant defense-related genes were depressed in the proteome of LTH and up-regulated in the transcriptome. Our study reveals the molecular interactions between *M*. *oryzae* and rice in both the transcriptome and proteome.

## Materials and methods

Our study was performed in Shenyang City (Lat: 41°8' N, Long: 123°38' E), Liaoning Province. Chinese government gave us permission to use this paddy field for scientific research.

### Plant materials, fungal materials, and fungal infection

Two rice cultivars, the durable resistant rice variety Gangyuan8 (GY8) and the susceptible rice variety Lijiangxintuanheigu (LTH), were grown in Shenyang City (Lat: 41°8' *N*, Long: 123°38' *E*), Liaoning Province, China. Each cultivar consisted of two m rows of 20 plants with 30 cm spacing between the rows. At 30 days after transplanting, the seedlings were spray-inoculated with a mixture of five isolates (ZB1, ZB13, ZC1, ZE1, and ZF1) that were isolated from the field in 2012 [25]. Leaves were harvested at 0 hpi (hours post inoculation) and 24 hpi for iTRAQ and RNA sequencing (RNA-Seq). There were two independent biological replicates in the iTRAQ experiment, and thus eight samples were included: GY8 at 0 hpi (GY8-0-1 and GY8-0-2), GY8 at 24 hpi (GY8-24-1 and GY8-24-2), LTH at 0 hpi (LTH-0-1 and LTH-0-2), and LTH at 24 hpi (LTH-24-1 and LTH-24-2). Four samples were used in the RNA-Seq, which included GY8 at 0 hpi (GY8-0), GY8 at 24 hpi (GY8-24), LTH at 0 hpi (LTH-0), and LTH at 24 hpi (LTH-24). Leaves were harvested at 0 hpi, 12 hpi, 24 hpi, 36 hpi, 48 hpi, and 60 hpi for western blotting (WB) or quantitative real-time PCR (qRT-PCR) experimentation, and there were three technical replicates in qRT-PCR and mixed samples and no replicates in WB. The details of sample design in every experiment were shown in S1 Table. All the samples were immediately frozen in liquid nitrogen and kept at -80°C until protein or RNA extraction. To ensure that the inoculation was successfully performed, the remaining seedlings were kept for another seven days post-inoculation for disease evaluation.

### Fungal growth on the rice sheath

GY8 and LTH were grown in a growth chamber at 28°C in a 12-h light/12-h dark photoperiod with 75% humidity. For microscopic monitoring of fungal development, a mixture of five isolates of the *M*. *oryzae* strain described above was used to inoculate the detached rice sheaths from 4-week-old rice plants. Spores were collected via flooding of the fungal agar cultures with sterile water, and the spore concentration in the suspension was adjusted to approximately 5 × 105 conidia/ml. The detached rice sheath assay was performed as described previously [16, 26]. All images were recorded using confocal microscopy (Nikon eclipse 80i, Nikon, Japan).

### Protein preparation

The samples were ground into powder in liquid nitrogen and extracted with lysis buffer (7 M urea, 2 M thiourea, 4%CHAPS, 40 mM Tris-HCl, pH 8.5) containing 1 mM phenylmethylsulphonyl fluoride (PMSF) and 2 mM ethylenediaminetetraacetic acid (EDTA). After 5 min, 10 mM dithiothreitol (DTT) was added to the samples. The suspension was sonicated at 200 W for 15 min and then centrifuged at 4°C and 30,000×*g* for 15 min. The supernatant was combined with 5×volume of chilled acetone containing 10% (v/v) trichloroacetic acid (TCA) and incubated at -20°C overnight. After centrifugation at 4°C and 30,000×*g*, the supernatant was discarded. The precipitate was washed with chilled acetone three times. The pellet was air-dried and dissolved in lysis buffer. The suspension was sonicated at 200 W for 15 min and centrifuged at 4°C and 30,000×*g* for 15 min. The supernatant was transferred to another tube. To reduce disulfide bonds in the proteins of the supernatant, 10 mM DTT was added and incubated at 56°C for 1 h. Subsequently, 55 mM iodoacetamide (IAM) (final concentration) was added to block the cysteines, following which the mixture was incubated for 1 h in a darkroom. The supernatant was mixed well with 5× volume of chilled acetone for 2 h at -20°C to precipitate the proteins. After centrifugation at 4°C and 30,000×*g*, the supernatant was discarded, and the pellet was air-dried for 5 min, dissolved in 500 μL 0.5 M triethylammonium bicarbonate (TEAB) (Applied Biosystems, Milan, Italy), and sonicated at 200 W for 15 min. Finally, samples were centrifuged at 4°C and 30,000×*g* for 15 min. The supernatant was transferred to a new tube and quantified. The proteins in the supernatant were kept at -80°C for further analysis.

### iTRAQ labeling and strong cation exchange fractionation

Total protein (100 μg) was collected from each sample solution and then digested with Trypsin Gold (Promega, Madison, WI, USA) at the ratio of protein: trypsin = 30: 1 at 37°C for 16 h. Following trypsin digestion, the peptides were dried by vacuum centrifugation. The peptides were reconstituted in 0.5 M TEAB and processed according to the manufacturer’s protocol for 8-plex iTRAQ reagent (Applied Biosystems). Briefly, one unit of iTRAQ reagent was thawed and reconstituted in 24 μL isopropanol. The peptides were labeled with the isobaric tags and incubated at room temperature for 2 h. The labeled peptide mixtures were then pooled and dried by vacuum centrifugation.

SCX chromatography was performed with a LC-20AB high-performance liquid chromatography (HPLC) pump system (Shimadzu, Kyoto, Japan). The iTRAQ-labeled peptide mixtures were reconstituted with 4 mL buffer A (25 mM NaH2PO4 in 25% ACN, pH 2.7) and loaded onto a 4.6 × 250 mm Ultremex SCX column containing 5-μm particles (Phenomenex). The peptides were eluted at a flow rate of 1 mL/min with a gradient of buffer A for 10 min, 5%–60% buffer B (25 mM NaH2PO4, 1 M KCl in 25% ACN, pH 2.7) for 27 min, and 60%–100% buffer B for 1 min. The system was then maintained at 100% buffer B for 1 min before equilibrating with buffer A for 10 min prior to the next injection. Elution was monitored by measuring the absorbance at 214 nm, and fractions were collected every 1 min. The eluted peptides were pooled into 20 fractions, desalted with a Strata X C18 column (Phenomenex), and vacuum dried.

### Liquid chromatography-electrospray ionization-tandem mass spectrometry (LC-ESI-MS/MS) analysis using a triple time-of-flight (TOF) 5600

Each fraction was resuspended in buffer A (5% ACN, 0.1% FA) and centrifuged at 20,000×*g* for 10 min. The final concentration of each peptide was about 0.5 μg/μL on average. Ten microliters of supernatant were then loaded onto a LC-20AD nano-HPLC (Shimadzu, Kyoto, Japan) by the autosampler onto a 2 cm C18 trap column. The peptides were then eluted onto a 10 cm analytical C18 column (inner diameter 75 μm) packed in-house. The samples were loaded at 8 μL/min for 4 min, following which a 35 min gradient was run at 300 nL/min starting from 2 to 35% B (95%ACN, 0.1%FA), followed by a 5 min linear gradient to 60% B and a 2 min linear gradient to 80% B where it was held for 4 min, finally returning to 5% over 1 min.

Data acquisition was performed on a TripleTOF 5600 System (AB SCIEX, Concord, ON, Canada) fitted with a Nanospray III source (AB SCIEX) and a pulled quartz tip as the emitter (New Objectives, Woburn, MA, USA). Data were acquired using an ion spray voltage of 2.5 kV, curtain gas of 30 psi, nebulizer gas of 15 psi, and an interface heater temperature of 150°C. For Information Dependent Acquisition (IDA), survey scans were acquired in 250 ms, and as many as 30 product ion scans were collected if they exceeded a threshold of 120 counts per second (counts/s) and with a 2+ to 5+ charge-state. The total cycle time was fixed to 3.3 s. The Q2 transmission window was 100 Da for 100%. Four time-bins were summed for each scan at a pulse frequency value of 11 kHz through the monitoring of the 40 GHz multichannel time-to-digital converter (TDC) detector with a four-anode channel detect ion. A sweeping collision energy setting of 35 ±5 eV coupled with iTRAQ adjust rolling collision energy was applied to all precursor ions for collision-induced dissociation. Dynamic exclusion was set for 1/2 of peak width (15 s), and then the precursor was refreshed off the exclusion list.

### Data analysis

Raw data files acquired from TripleTOF 5600 System were converted into MGF files using Proteome Discoverer 1.2 (Thermo Electron Corporation, Massachusetts, US) and 5600 MS converter, and the MGF files were searched. Protein identification was performed using the Mascot search engine (Matrix Science, London, UK; version 2.3.02) against a database containing rice sequences.

For protein identification, a mass tolerance of 0.05 Da was permitted for intact peptide masses and 2 PPM for fragmented ions, with allowance for one missed cleavage in the trypsin digests. Gln->pyro-Glu (N-term Q), Oxidation (M), and Deamidated (NQ) were the potential variable modifications, and Carbamidomethyl (C), iTRAQ8plex (N-term), and iTRAQ8plex (K) were the fixed modifications. The charge states of the peptides were set to +2 and +3. Specifically, an automatic decoy database search was performed in Mascot by choosing the decoy checkbox in which a random sequence of the database is generated and tested for raw spectra as well as the real database. To reduce the probability of false peptide identification, only peptides at the 95% confidence interval identified by Mascot probability analysis as greater than “identity” were counted as identified. Each confident protein identification involved at least one unique peptide.

### Criteria for identifying differentially expressed proteins

To identify the induced expressed incompatible interaction (GY8) and compatible interaction (LTH) proteins, fold change (FC) of each biological repeat were calculated, and FC ≥ 1.2 or ≤ 0.833 was taken as the criteria significance of different express proteins. We chose genes that had the same trend of differentially expressed in the two replicates, and that had a significant level of differentially expressed in at least one repeat, as the differentially expressed genes. The FC values and significant levels of each repetition are detailed in S2 and S3 Tables.

### Functional method description

Functional annotations of the proteins were conducted using the Blast2GO program against the non-redundant protein database (Nr). The Kyoto Encyclopedia of Genes and Genomes (KEGG) database (http://www.genome.jp/kegg/) and the Gene Ontology (GO) knowledgebase (http://geneontology.org/) were used to classify and group these identified proteins. The web-tool STRING 11.0 (http://string-db.org) was used to analyze the protein-protein interaction.

### Western blotting analysis

To validate the reliability of the iTRAQ data, the rice leaves described above (2.1) were used to monitor the expression patterns by western blotting. The antibody was a rabbit polyclonal antibody generated using a synthetic peptide as the immunogen and was produced by BPI (Beijing Protein Innovation Co., Ltd., Beijing, China). For sodium dodecyl sulfate-polyacrylamide gel electrophoresis (SDS-PAGE), 50 μg of each sample were separated by 5% stacking gel (5% Acr-Bis, 0.125 M Tris-HCl pH 8.8, 0.015% SDS, 0.015% ammonium persulfate, 0.006% TEMED) at 60 V for 40 min and electro-transferred onto a NC/PVDF membrane (Millipore Corporation, Bedford, MA, USA) at 200 mA for 60–120 min. The membrane was immersed in 5% nonfat milk TBST solution (200 mM Tris-HCl, 1.5M NaCl, 0.5% Tween-20, pH 7.5) at room temperature for 1.5–2 h, following which it was incubated with the primary antibodies in 5% nonfat milk TBST at 4°C for 12 h and rinsed three times by TBST solution. The membrane was then incubated with secondary antibodies in 5% nonfat milk TBST at room temperature for 1 h and rinsed three times with TBST solution. The blot was developed with an ECL Plus kit (Millipore Corporation, Bedford, MA, USA) according to the manufacturer’s instructions. The images scanned were directly performed by Odyssey^®^ CLx Imaging System (Li-COR, Los Angeles, USA). The antibodies used in the western blotting are described in S4 Table. We use Gel-Pro Analyzer 4 software (Media Cybernetics, Maryland, US) to analyze the grayscale of each band of gel image, use the grayscale as the expression of the protein. Then we calculated gray ratio of detected gene and reference gene as the relative expression.

### RNA extraction and RNA-Seq analysis

Total RNA was extracted from the rice leaves described above (2.1) using TRIzol^®^ reagent (Ambion TM, Lot No. 15596018) according to the manufacturer’s protocol, and the RNA samples were treated with DNase I (TaKaRa, Japan) for 4 h. The cDNA library preparation and sequencing reactions were conducted at the Biomarker Technology Company, Beijing, China. RNA-Seq analysis was performed as described by Zheng [27]. The cDNA library was sequenced on the Illumina Cluster Station and Illumina Genome Analyzer system. Reads from each library were assembled separately. The Trinity method [28] was used for *de novo* assembly of Illumina reads of hawthorn (*Crataegus* sp.).

We annotated the unigenes based on a set of sequential BLAST searches [29] designed to find the most descriptive annotation for each sequence. The assembled unigenes were compared with sequences in the National Center for Biotechnology Information (NCBI) Nr protein and nucleotide (Nt) databases (https://www.ncbi.nlm.nih.gov/), the UniProt database (https://www.uniprot.org/), the KEGG pathway database (http://www.genome.jp/kegg/), and the COG database (http://www.ncbi.nlm.nih.gov/COG/). The Blast2GO program [30] was used to obtain Gene Ontology (GO) annotations of the unigenes. WEGO software (http://wego.genomics.org.cn/) was then used to perform GO functional classification of all unigenes to view the distribution of gene functions.

Gene expression levels were measured in the RNA-Seq (Invitrogen) analyses as numbers of reads normalized by Reads Per Kilobase of transcript per Million mapped reads (RPKM). IDEG6 software [31] was used to identify DEGs in pairwise comparisons, and the results of all statistical tests were corrected for multiple testing with the Benjamini–Hochberg false discovery rate (FDR <0.01). Sequences were deemed to be significantly differentially expressed if the adjusted *P*-value obtained by this method was <0.001 and there was at least a FC (>1 or <− 1 in log _2_ FC) in RPKM between two libraries.

### qRT-PCR

To confirm the results of the RNA-Seq analysis, the rice leaves described above (2.1) were used to monitor the expression levels of eight DEGs by qRT-PCR. RNA extraction was as described in 2.8. The cDNA was synthesized using an RNA reverse-transcription kit (Invitrogen Life Technologies, Shanghai, China). The concentration of each RNA sample was adjusted to 1 mg/mL with nuclease-free water, and total RNA was reverse-transcribed in a 20 mL reaction system using the AMV RNA PCR Kit (TaKaRa). The qPR-PCR was carried out on the LightCycler 480@ II with LightCycler 480@ SYBR I Master (Roche Applied Science, Basel, Switzerland) under the following conditions: 95°C for 5 min, followed by 50 cycles of 95°C for 10 s, 60°C for 15 s, and 72°C for 20 s, followed by melting curve generation (68°C to 95°C). Primers used in the qRT-PCR for validation of DEGs are shown in S5 Table. OsActin was used as the internal control, and the 2^–ΔΔC^_T_ method was used to calculate relative expression levels [32].

## Results

### Differences in *M*. *oryzae* development between GY8 and LTH within 48 hpi

**Fig 1:**
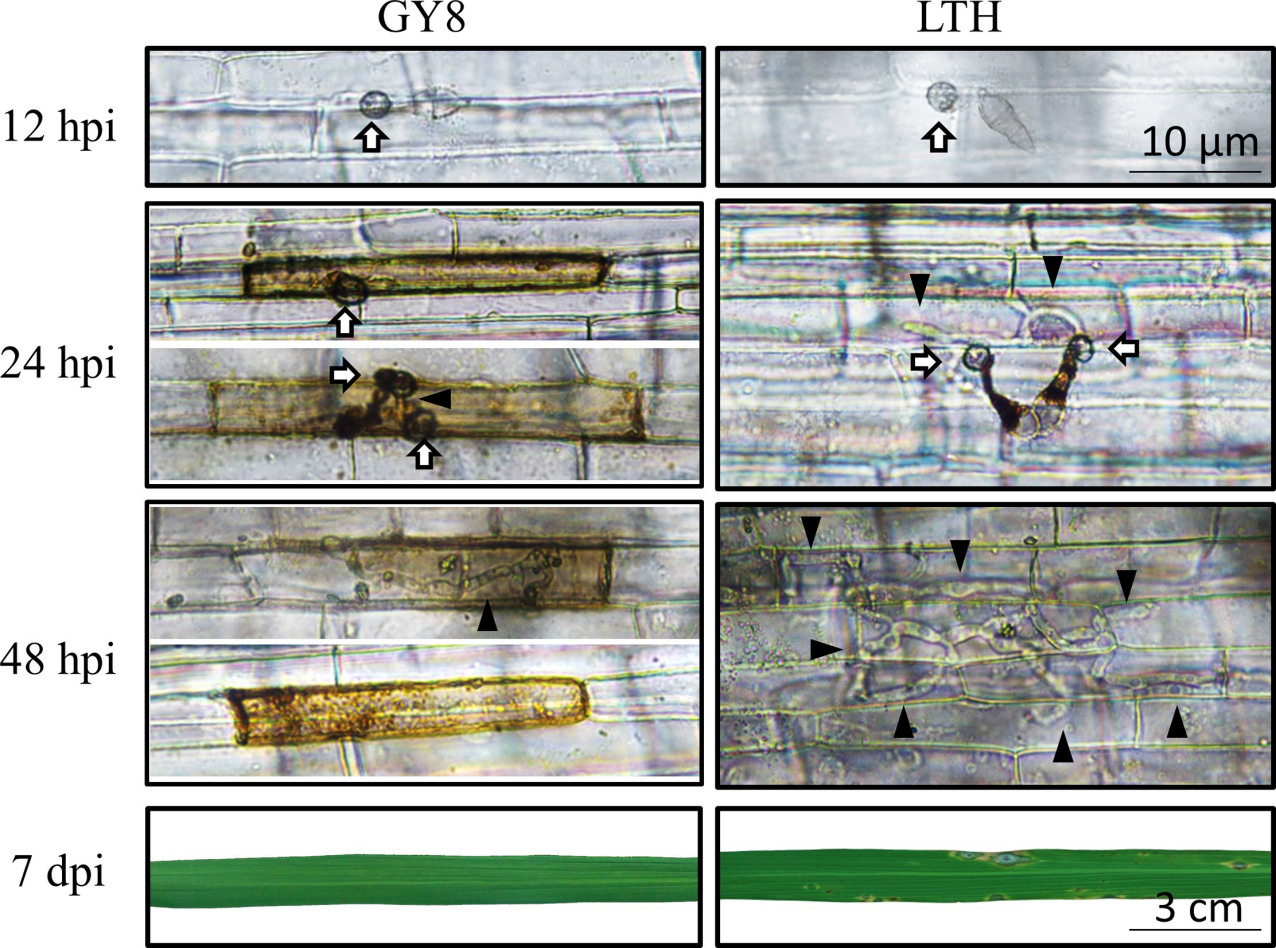
Development of *Magnaporthe oryzae* on rice leaf sheaths post-inoculation and the leaf at 7 d post-inoculation. The leaf sheaths of the durable resistant rice Gangyuan8 (GY8) and susceptible rice Lijiangxintuanheigu (LTH) were inoculated with spore suspensions of *M*. *oryzae*. The inoculated leaf sheaths were examined under a microscope at the time points of 12 hpi (hours post-inoculation), 24 hpi, and 48 hpi, and the leaves were photographed at 7 dpi (days post-inoculation). Arrows indicate infection structure appressorium, and the black triangle indicates invasive hyphae.

To explore the stage at which GY8 prevents the invasion of *M*. *oryzae*, we examined the development of *M*. *oryzae* during the first 48 hpi on the durable resistant rice GY8 and the susceptible rice LTH. To clearly observe this, we used rice sheath tissue to microscopically observe the penetration of *M*. *oryzae* in the rice cuticle, as this tissue has less chlorophyll than the green leaves. By 12 hpi, appressorium formation was evident as a dome-shaped structure at the tip of the germ tube of *M*. *oryzae* on both GY8 and LTH (Fig 1). These results demonstrate that before the penetration of the host cuticle, the early development of *M*. *oryzae* is similar between GY8 and LTH, including conidial germination, germ tube extension, appressorium formation, and maturation.

We found significant differences in the development of *M*. *oryzae* at 24 hpi on GY8 and LTH. At 24 hpi, GY8 displayed programmed cell death, while the penetration peg and primary invasive hyphae emerged from the appressorium on LTH. At 48 hpi, the secondary invasive hyphae of *M*. *oryzae* on the LTH sheath expanded into the neighboring rice cells. In striking contrast, *M*. *oryzae* on GY8 only showed the initial emergence of a short penetration peg.

### Identification of DEPs induced by *M*. *oryzae* invasion

**Fig 2:**
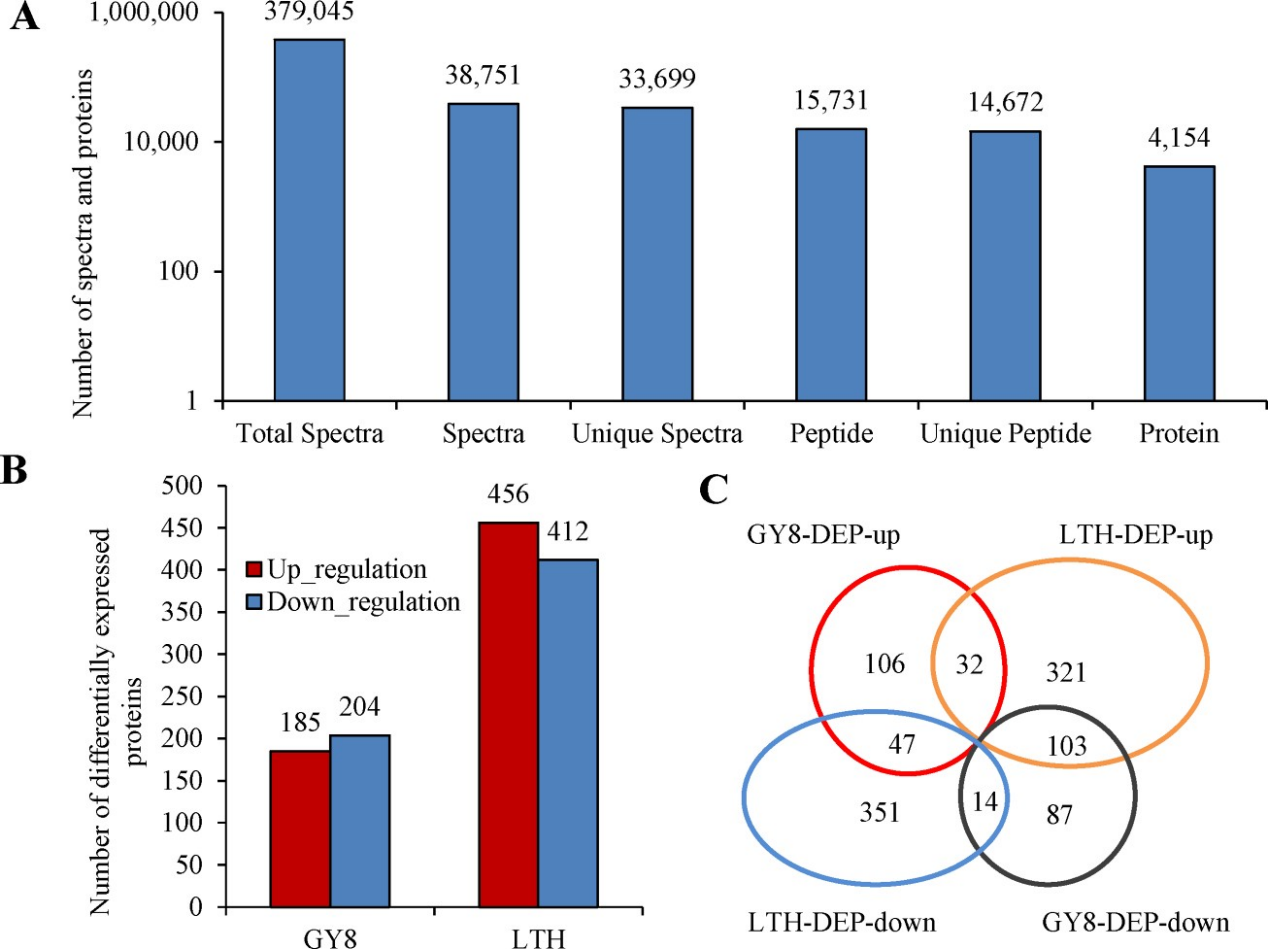
Distribution of differentially expressed proteins (DEPs) between the resistant (GY8) and susceptible (LTH) variety following inoculation with *M*. *oryzae*. A: Results of the mass spectrometry analysis and protein identification. B: Number of up-regulated and down-regulated proteins. C: Venn diagram analysis the DEPs that were up- or down-regulated in GY8 and LTH.

The iTRAQ technique was used to contrast the differences in the proteome between the compatible and incompatible reactions induced by *M*. *oryzae* invasion. After analysis by Mascot software, a total of 379,045 spectra were detected, including 38,751 identified spectra and 33,699 unique spectra, of which 15,731 spectra could be matched to peptides in the database and 14,672 were unique peptides (Fig 2A). In total, 4,154 proteins were identified using the *Oryza sativa* UniProt database with an FDR <0.01, as stated in the methods.

A total of 389 and 868 DEPs were identified in GY8 and LTH (Fig 2B), of which 196 were shared between GY8 and LTH, and 193 were GY8-specific and 672 were LTH-specific (Fig 2C). The list of differentially expressed proteins induced by M. oryzae invasion in GY8 and LTH were shown in S2 and S3 Tables. In detail, among the GY8-specific DEPs, 106 were up-regulated and 87 were down-regulated. Among the LTH-specific DEPs, 321 were up-regulated and 351 were down-regulated. Among the shared DEPs, 46 were similarly regulated between GY8 and LTH, whereas 150 were differentially regulated. These results suggest that most DEPs are either GY8- or LTH-specific.

### GO analysis of DEPs

The PANTHER (Protein Analysis through Evolutionary Relationships) Classification System (http://pantherdb.org/) was used to classify the proteins of GY8 and LTH induced by *M*. *oryzae*.

As was shown in S1 Fig, the DEPs of GY8 and LTH were all classified into three GO categories, including 22 functional categories. There were eight GO terms in Biological Process, eight in Molecular Function, and six in Cellular Component. Proteins involved in GY8 and LTH interactions with rice blast mainly concentrated in organelle, cell part, catalytic activity, binding and metabolic process. In the biological process, most proteins were assigned to cellular process, which indicated that the enzyme catalysis, cell components and intracellular metabolic activity were closely related to the response of rice to blast fungus. As the total number of DEPs obtained from GY8 was small, the enriched genes in each category of GY8 were less than LTH, but the proportions of genes enriched in each GO classification were similar in GY8 and LTH.

### Specific metabolic processes in GY8 and LTH are involved in the response against *M*. *oryzae*

To identify specific metabolic processes in GY8, we used the KEGG database to analyze the DEPs in GY8 and LTH following *M*. *oryzae* inoculation. We found that many proteins involved in plant-pathogen interaction (Ko04626), plant hormone signal transduction (Ko04075), fatty acid metabolism (Ko00071), and peroxisome (ko04146) were specifically activated or unchanged in GY8 but were inhibited in LTH during the rice–*M*. *oryzae* interaction (S6 Table).

We found 11 DEGs enriched in plant-pathogen interaction. Among them, the FLS2-like protein was up-regulated in GY8 and down-regulated in LTH. One OsCEBiP and three heat shock protein (HSP90) proteins were unchanged in GY8 but down-regulated significantly in LTH. Additionally, two Ca^2+^-dependent protein kinases (CDPK) and one CaMCML protein were specifically up-regulated in LTH but unchanged in GY8, which implied that these proteins may negatively modulate blast resistance in GY8 and LTH [33].

In the plant hormone signal transduction pathway, we found that one cytokinin (CK) receptor-like protein (Os07g0539900) was up-regulated in GY8 and unchanged in LTH, while two membrane brassinosteroid (BR) receptor-like proteins, two CK receptor-like proteins, and three gibberellin (GA) receptor Gid1-like proteins were down-regulated in LTH but were unchanged in GY8. In contrast, an OsGH3 (Os07g0592600) encoding enzyme catalyzing auxin indoleacetic acid (IAA) and one abscisic acid (ABA) receptor OsPYL10 (Os10g0573400) were found to be restrained in GY8 but activated in LTH. The result implied that BR, CK, and GA positively modulate rice blast resistance, while ABA and IAA demonstrated the opposite, which is in agreement with research that exogenous ABA decreases resistance to blast disease [33].

After inoculation, seven DEPs were detected to be involved in fatty acid metabolism in GY8 and LTH. Five of these were significantly down-regulated in LTH, while the expression abundance was not significantly changed in GY8. Three of the five proteins (Os02g0817700, Os10g0457600, and Os06g0103500) are involved in the oxidation of fatty acids in the redox pathway.

### Expression of putative rice blast pathogenesis-related (PR) proteins and receptor kinases (RKs)

PR proteins are known to participate in the growth inhibition of a variety of pathogenic bacterial and fungal strains and are thought to be useful for the development of antimicrobial agents [34]. We investigated the expression of PR proteins and their roles in the rice defense response to *M*. *oryzae*. The gene IDs and family names were obtained according to Dou [35], and the gene name was referenced from the Rice Annotation Project database (RAP-DB https://rapdb.dna.affrc.go.jp/index.html#).

**Fig 3:**
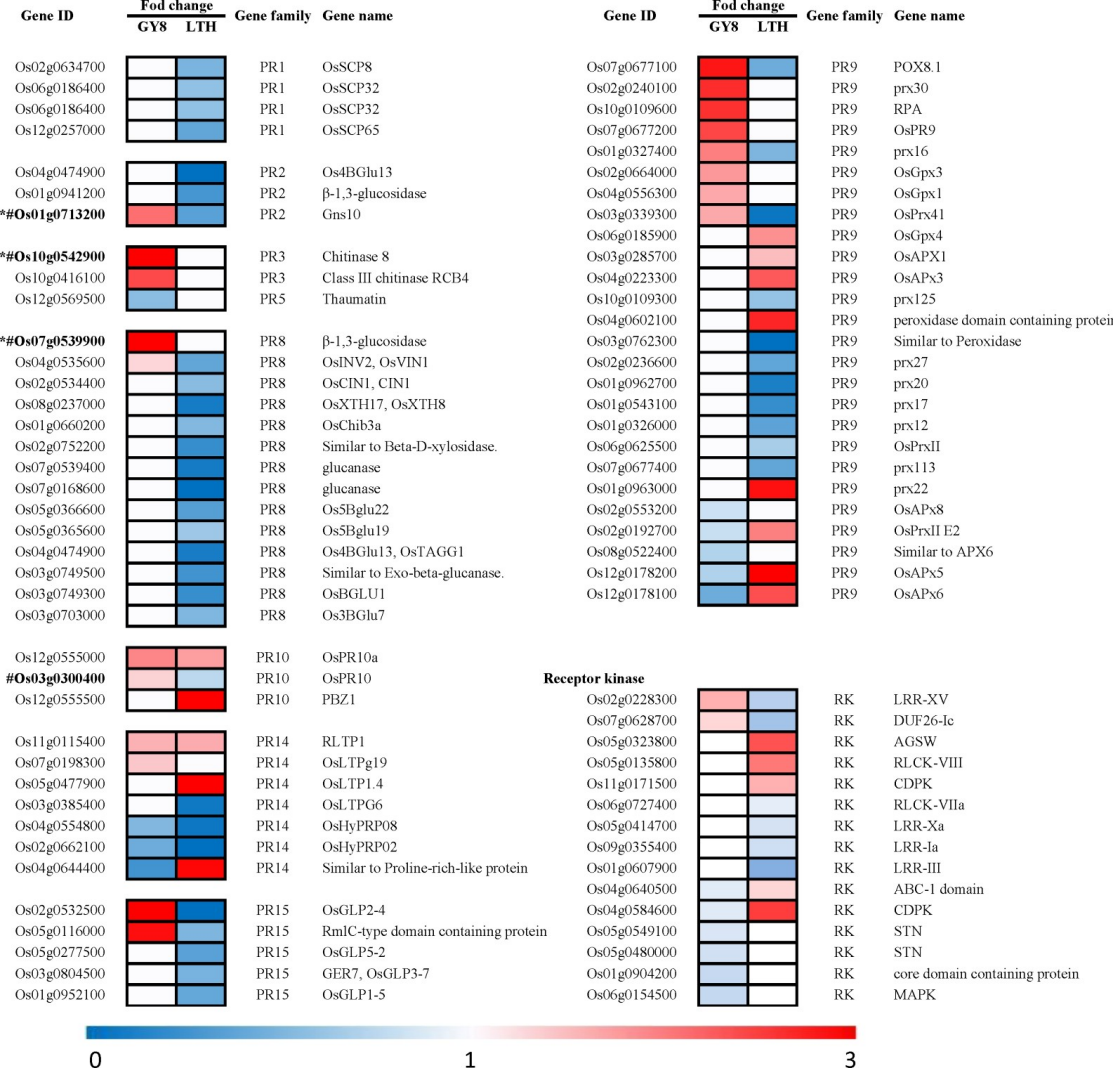
Heat maps showing the expression patterns of pathogenesis-related (PR) and receptor kinase (RK) genes that were identified as differentially regulated in durable resistant and susceptible rice by iTRAQ. The gene IDs and family names of the pathogenesis-related genes were obtained according to Dou [35]. The IDs of the RK genes were retrieved from the rice kinase database [36]. The gene names of the DEGs were obtained from the Rice Annotation Project Database (RAP-DB). The fold-change color of each gene represents the expression pattern of this gene. Red represents a fold-change greater than 1, indicating that this gene was up-regulated at 24 h post rice blast inoculation, and the lighter the color, the lower the up-regulated expression level. White represents a fold-change equal to 1, indicating no change, while blue represents a lower level of down-regulated expression with a darker color. See the scale at the bottom of the figure for details. *The transcript expression pattern of this gene was verified by qRT-PCR. ^#^ The protein expression pattern of this gene was verified by western blotting.

As shown in Fig 3, 66 PR proteins that were distributed across nine PR gene families were significantly differentially expressed in GY8 or LTH following inoculation with *M*. *oryzae*. Thirty of these PR proteins belong to PR1, PR2, PR3, PR8, and PR15 and were down-regulated in LTH but remained unchanged in GY8. Fifteen PR proteins, including serine carboxypeptidase-like proteins (SCPLs), serine carboxy peptidase (SCP), β-1,3-glucanse, and chitinase (CHT), were down-regulated in LTH but up-regulated or unchanged in GY8. Conversely, eight peroxidases were induced to be up-regulated in LTH, while the expression was inhibited or unchanged in GY8.

According to the results, the defense response occurred in both GY8 and LTH, but the number of PR proteins with up-regulated expression was higher in GY8 than in LTH, and most of them have been reported to be involved in general fungal defense responses, such as pathogen recognition, signal transduction, and reactive oxygen burst. After pathogen immunity is initiated by plants, a series of signal transduction pathways will be activated and a cross-linked signal network will be formed, among which kinase-mediated phosphorylation is one of the most important pathways [37]. We found that two protein kinases (Os02g0228300 and Os07g0628700) were specifically up-regulated in GY8 in response to *M*. *oryzae*, and five RKs were up-regulated in LTH and down-regulated in GY8. In addition, we also found eight receptor protein kinases that were down-regulated exclusively in GY8 or LTH. The gene IDs and family were retrieved from the rice kinase database [36].

### Proteins networks analysis

**Fig 4:**
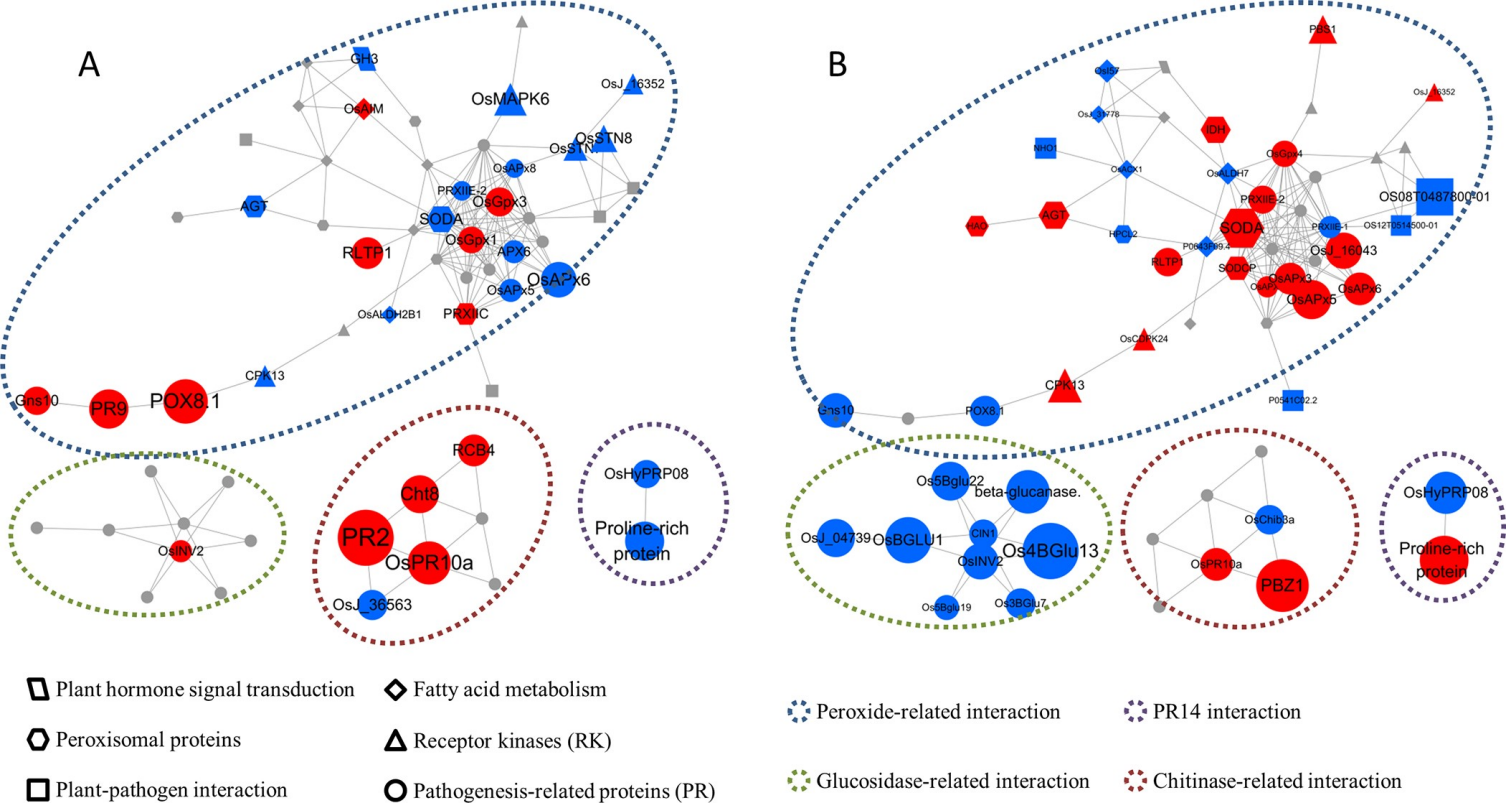
Interaction network of crucial DEPs in GY8 and LTH. A: Interaction network of DEPs in GY8; B: Interaction network of DEPs in LTH. The color of spot represents the expression pattern. Red represents up-regulated, blue represents down-regulated and gray represents unchanged. The size of spot represents the fold-change, the bigger the size, the higher the fold-change. The shape represents the family or pathway of the protein. Four main clusters were circled by broken Line with different color.

As was mentioned above, we found 105 proteins may play important roles at incompatible interaction (GY8). These proteins involved in plant-pathogen interaction, plant hormone signal transduction, fatty acid metabolism, peroxisome biosynthesis or belonged to PR or RK. To identify the interactions between these proteins, the web-tool STRING 11.0 (http://string-db.org) was used to analyze the protein-protein interaction (PPI), and 61 proteins were implicated in “peroxide”, “Glucosidase”, “Chitinase”, and “PR14 gene family” functional clusters. As can be seen from Fig 4, in the “peroxide-related interation” cluster, a lot of proteins were down-regulated in GY8, but up-regulated or unchanged in LTH, such as SODA、AGT、CDK13、OsMAPK6、OsSTN、OsJ-16352、OsAPX et al.. They are related to peroxide signal transduction and biosynthesis. The differential expression of these proteins can significantly affect the hydrogen peroxide scavenging and post invasion defense of plants, which explained that the difference of programmed cell death phenomenon between GY8 and LTH.Glucan and chitin are the main structural component of cell wall of rice blast fungus and can be decomposed by Glucanase and chitinase. In the "Glucosidase" interaction group, the proteins in GY8 was up-regulated or unchanged at 24 hpi, while nine Glucanases were down-regulated in LTH, which might be caused by effectors that were released into cells, which leads to the PTI system failure. Similarly, in chitinase interaction groups, PR2, Cht8 and RCB4 were significantly up-regulated in GY8 after inoculation, but remained unchanged or down-regulated in LTH.

### Western blotting analysis

**Fig 5:**
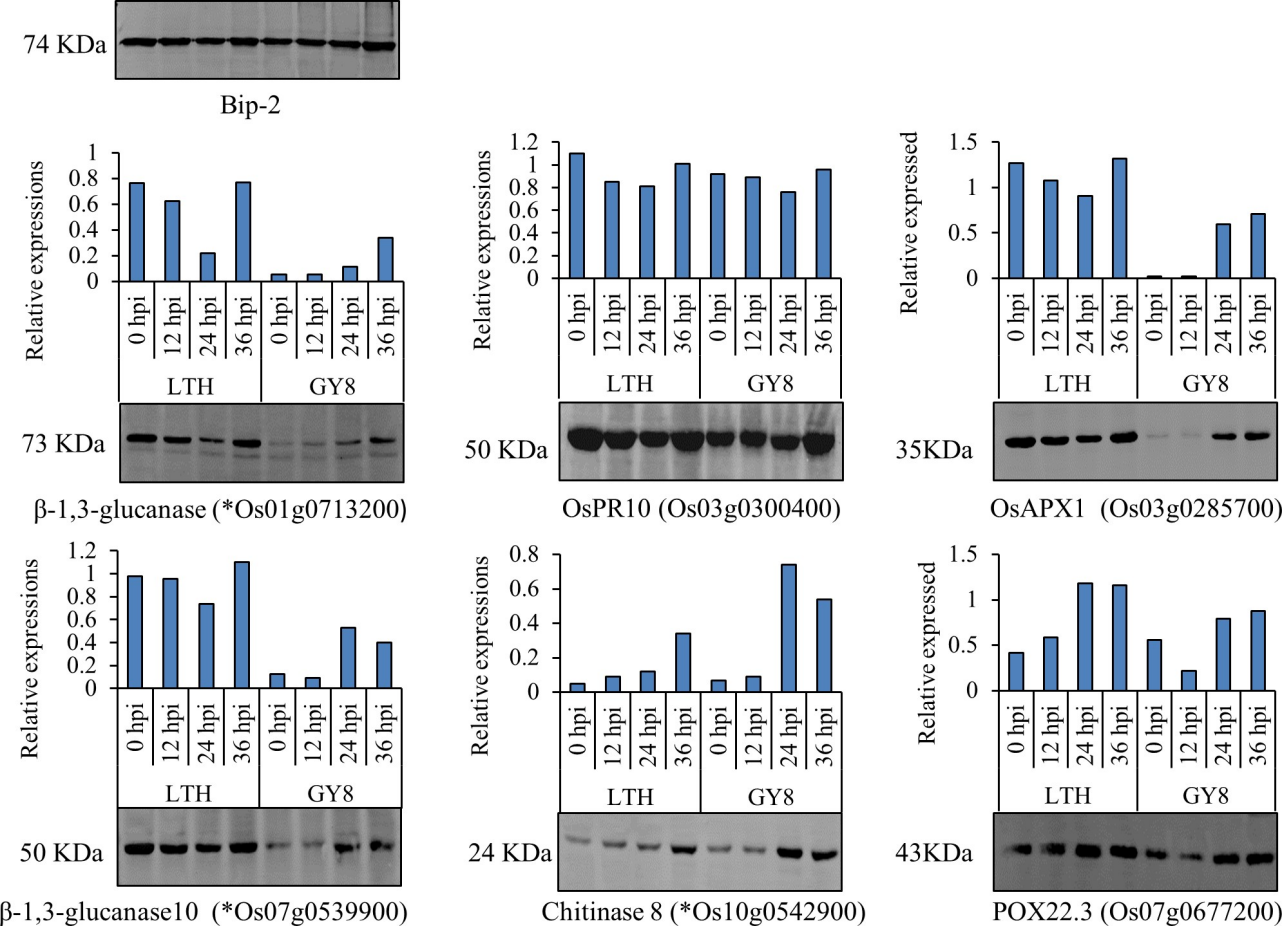
Results of the western blotting analyses. We use Gel-Pro Analyzer 4 software to analyze the grayscale of each band of gel image, use the grayscale as the expression of the protein. Then we calculated gray ratio of detected gene and reference gene (Bip-2) as the relative expression.

To confirm the result of the iTRAQ analysis and clarify the dynamic changes in the expression levels of disease-resistant proteins at different periods after inoculation, we conducted quantitative analysis of four proteins in the leaves of LTH and GY8 inoculated at 12 h, 24 h, and 36 h by western blot. The results showed that β-1,3-glucanase (Os01g0713200, PR2 family protein), and β-1,3-glucanase 10 protein (Os07g0539900) in LTH were all down-regulated at 12 h and 24 h after inoculation with rice blast fungus and remained unchanged at 36 h (Fig 5). However, the expression of these two proteins was up-regulated after GY8 was inoculated, especially at 24 h and 36 h, which agreed with the results of the iTRAQ analysis. Interestingly, the basal levels (0 h) of these two proteins in LTH before inoculation were much higher than that of GY8. The expression of OsPR10 (Os03g0300400) was similar in LTH and GY8, and the difference was not significant in the three time periods after inoculation and before inoculation. A Chitinase 8 (Os10g0542900) protein was strongly expressed in GY8 at 24 hpi, slightly decreasing at 36 h. The expression level of the Chitinase 8 protein also increased following LTH inoculation, but this was not as significant as in GY8. Two peroxisome (Os03g0285700 and Os07g0677200) protein were both up-regulation in GY8 and LTH as being inoculated by *M*. *oryzae*. However, the abundance of Os03g0285700 and Os07g0677200 in LTH was much higher than that of GY8.

### Complementary transcriptomics research by RNA-Seq

**Fig 6:**
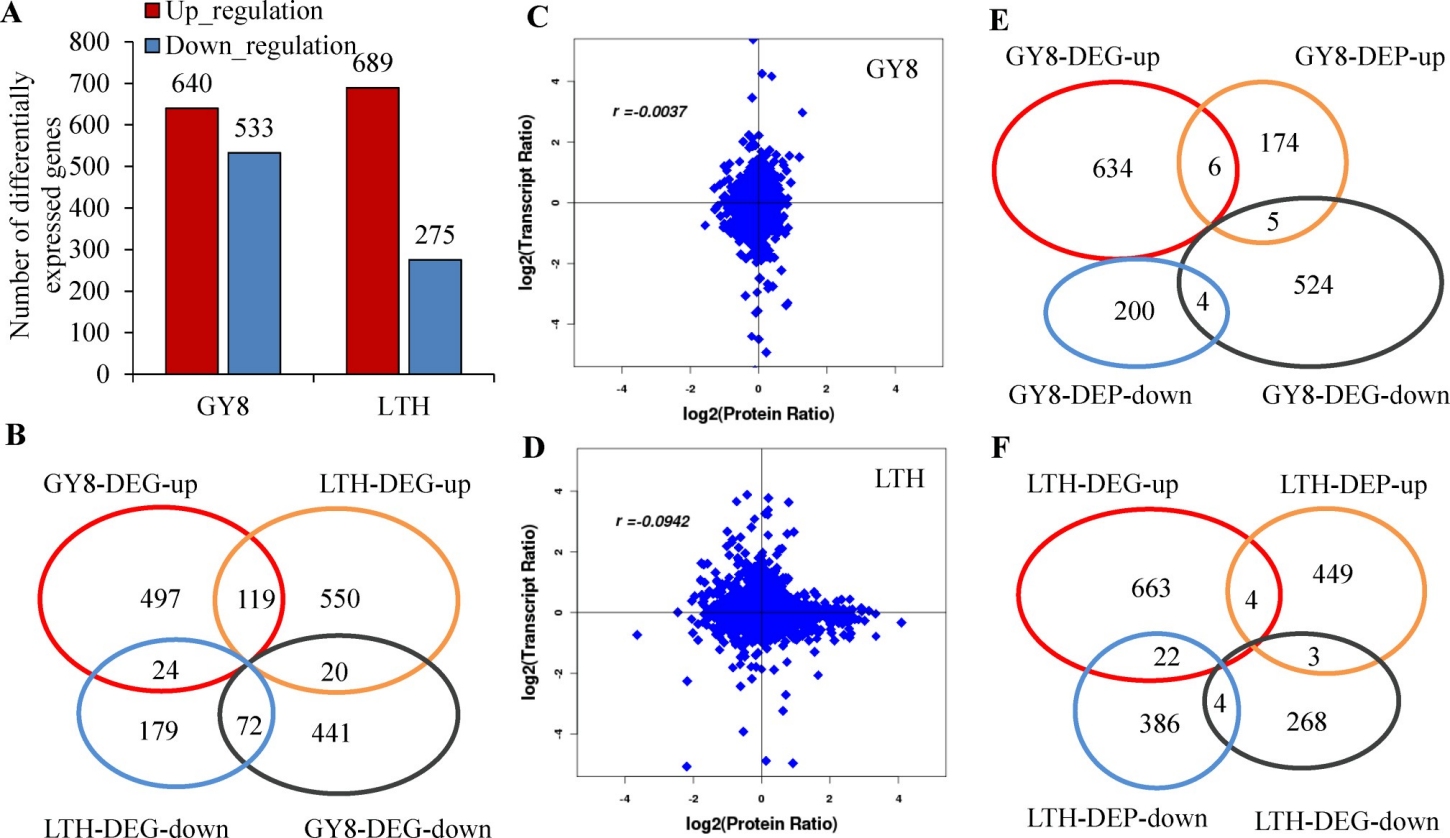
Distribution of differentially expressed genes (DEGs) between GY8 and LTH after inoculation with *M*. *oryzae*. A: The numbers of up-regulated and down-regulated genes in GY8 and LTH following *M*. *oryzae* infection; B: Venn diagram of DEGs between GY8 and LTH; C, D: Concordance between changes in and its encoded protein in the developing grains at four sampling times. Transcript ratio and Protein ratio, the fold changes of transcript and protein after inoculated with *M*. *oryzae*, in LTH and GY8, respectively. r, Pearson correlations coefficient of the comparisons between fold changes of proteins and transcripts; E: Venn diagram between DEPs and DEGs of LTH after inoculated with *M*. *oryzae*; F: Venn diagram between DEPs and DEGs of LTH after inoculated with *M*. *oryzae*.

Using iTRAQ technology, we identified a large number of DEPs related to signal recognition and conduction in disease-resistant varieties and susceptible varieties. TFs, as important components of rice disease resistance regulation, such as WRKY and C2H2, were not detected. To further confirm the proteomic analysis results, transcriptome sequencing was performed on the same samples. Genes whose expression levels increased or decreased by log2FC ≥1 or more at 24 hpi compared with 0 hpi were identified as differentially expressed. A total of 1173 and 964 DEGs were identified in GY8 and LTH, respectively, 235 of which were shared by GY8 and LTH, whereas 935 were GY8-specific and 729 were LTH-specific. The list of differentially expressed genes induced by M. oryzae invasion in GY8 and LTH were shown in S7 Table. Among the commonly shared DEGs, 191 were similarly regulated between GY8 and LTH, while 44 were differentially regulated (Fig 6A and 6B).

Similar to the results of the proteomic sequencing, many DEGs were enriched in the metabolic pathways of plant-pathogen interaction, plant hormone signal transduction, fatty acid metabolism, and peroxisome biosynthesis. Interestingly, most DEGs were specifically up-regulated in LTH, such as OsCEBiP, which was up-regulated in LTH, while the expression level in GY8 was not significantly changed after inoculation with *M*. *oryzae*. This result was contrary to that obtained by iTRAQ analysis (S8 Table). Compared with the differential proteins, the number of differential genes was greater, and more DEGs were found in GY8 than LTH, which is contrary to the results of the protein sequencing. Most importantly, we found fewer differentially expressed TF proteins from GY8 and LTH by iTRAQ analysis. However, according to the transcriptome sequencing results, a total of 92 differentially expressed TFs in the two varieties were detected after inoculation, which were summarized into 31 families, including WRKY, MYB, BHWH, and ERF. Among all the differentially expressed TFs, 40 were specific to GY8 (S9 Table).

From the Venn diagram and scatter diagram, it can be seen that the DEGs and DEPs in the two rice varieties were less correlated, and the fitting coefficients between them were 0.0039 and 0.0942 in GY8 and LTH, respectively (Fig 6C, 6D, 6E and 6F). Moreover, only nine DEGs and DEPs were differentially expressed in the same pattern at the transcriptome level and proteome level.

### Validation of DEGs by qRT-PCR

To verify the results of the transcriptome sequencing and identify the dynamic changes in gene expression at different times after inoculation, eight genes were selected for qRT-PCR at 0 hpi, 12 hpi, 24 hpi, 36 hpi, 48 hpi, and 60 hpi.

The transcriptome sequencing results showed that β-1,3-glucanase genes (Os01g0713200, Os07g0539100, Os07g0539900) and Chitinase genes (Os10g0542900, Os10g041600) were up-regulated in GY8 and LTH after inoculation with rice blast fungus. In the qRT-PCR analysis, after inoculation for 24 h, these five genes were up-regulated in both GY8 and LTH, which was completely consistent with the results of the transcriptome sequencing. Furthermore, we noticed that the expression levels of the five genes in GY8 at 12 h and 24 h after inoculation were significantly higher than that of LTH. Meanwhile, the five genes were almost down-regulated at 36 h, 48 h, and 60 h after being inoculated in the two varieties, which implied that 12 h and 24 h after inoculation are the key periods for rice to respond to *M*. *oryzae* infection [12]. It is noteworthy that, in the iTRAQ analysis, all five proteins were significantly up-regulated after GY8 inoculation for 24 h but down-regulated or not significantly changed in LTH.

The qRT-PCR results (S2 Fig) showed that wall-associated kinase (WAK) genes (Os02g0632800 Os09g0471500, and Os04g037500) were up-regulated in GY8 but down-regulated in LTH after inoculation for 12 h and 24 h. Meanwhile, in the iTRAQ analysis, there was no significant difference in the three proteins before and after inoculation in both GY8 and LTH.

## Discussion

### DEPs detected by iTRAQ in the durable resistant rice variety GY8 and the susceptible rice variety LTH in response to *M*. *oryzae* invasion

In previous studies, 2-DE has been widely used to identify proteins expressed in rice tissues in response to the rice blast fungus or some signaling molecules such as jasmonic acid [18]. Dozens of DEPs have been found [19, 20], such as PBZ1, OsPR-10, POX22.3, Glu1, Glu2, TLP, OsRLK, and Salt proteins, which were induced significantly in incompatible reactions. The early and high induction of these genes may enable the host plants to defend themselves [20].

In this research, 389 and 868 DEPs were found in GY8 and LTH, respectively, using the iTRAQ technique. All previously reported DEPs were detected in this research. As observed in a previous study [38], many PR proteins, particularly β-glucosidase and chitinase, were specifically up-regulated in the resistant variety GY8. Contrary to the findings of this previous study, PBZ1 and phenylpropanoid proteins, which have been reported to be related to plant defense, were up-regulated in both GY8 and LTH, and the degree of expression in LTH was even higher than that in GY8, which indicated that these proteins were not directly related to rice defense to *M*. *oryzae* in GY8. In addition to all the previously reported proteins, numerous other DEPs were found that are involved in plant-pathogen interaction, plant hormone signal transduction, fatty acid metabolism, and peroxisome, such as the OsFLS2-like protein, OsCEBiP, HSPs, peroxidases, CK receptor-like proteins, BR receptor-like proteins, GA receptor Gid1-like proteins, and the ABA receptor OsPYL10. In addition, we also discovered that some receptor protein kinases, such as OsCERK1 and CPKs, were involved in defense against *M*. *oryzae* in GY8.

### Proteins involved in the defense response were repressed in the susceptible rice variety LTH

Rice PTI immune systems against pathogens are mediated by LysM motif-containing proteins CEBiP [39], OsLYP4, OsLYP6 [9], OsFLS2 [40], and XA21 [41]. The receptor proteins perceive chitin molecules and flagellin flg22 derived from pathogens [9, 42] and then initiate immune signaling. The signal is then transmitted downstream through an MAPK cascade, leading to the activation of the immune response. When attacked by pathogens, receptor genes and MAPK cascade genes in rice cells are activated rapidly [43]. Many previous studies have confirmed that the expression of disease-resistant proteins and disease-resistant genes was up-regulated in both disease-resistant and susceptible varieties, but the up-regulated expression was earlier and higher in disease-resistant varieties [12–17]. Using qRT-PCR in the present study, it was also found that the genes encoding PR1, PR10, chitinase, and glucanase were induced to express early and at a high level in the disease-resistant varieties (S2 Fig). However, at the protein level, we found different results from the transcriptome sequencing and qRT-PCR. We noticed that most DEPs involved in PAMP recognition were significantly down-regulated in LTH and unchanged in GY8, such as CEBiP, OsFLS2-like protein, HSP90, and many proteins in the auxin metabolic pathway and most of the PR proteins. More specifically, many disease-resistant proteins were highly expressed in the susceptible variety before inoculation, but were inhibited after inoculation, while the basic expression of such proteins in the resistant variety was not high, but the expression was significantly up-regulated after induction by pathogens. Thus, in researching disease-resistance mechanisms, we should not only focus on the proteins that induce up-regulated expression but also on the proteins that are down-regulated in the susceptible varieties but maintain the same expression level in the resistant varieties. This would provide references for the in-depth analysis of different disease-resistance mechanisms.

### Comparison of transcript and protein levels

Many previous studies have found a low correlation between transcripts and proteins, such as the study on *Synechocystis* under salt stress and nitrogen starvation, indicating divergent regulation of transcriptional and post-transcriptional processes [44, 45]. In the present study, the fitting coefficient of the DEPs and DEGs was small in the correlation analysis. However, further analysis revealed that although fewer genes were detected by both methods, the enriched metabolic pathways were consistent. Additionally, it was found that more WRKY, C2H2, and other TFs were detected in the transcriptome than in the proteome, indicating that these regulatory factors play a role at the transcription level (S9 Table).

The results of transcriptome sequencing, iTRAQ analysis, qRT-PCR, and western blotting showed that three genes belonging to the PR2, PR3, and PR8 families, encoding chitinase and glucanase, were significantly up-regulated at both the transcriptome and protein levels 24 h after inoculation in GY8. However, in LTH, the transcriptome was significantly up-regulated 24 h after inoculation, while protein expression was significantly down-regulated or unchanged. In addition, many genes in LTH were up-regulated at the transcriptome level but down-regulated at the protein level. These genes are typically associated with disease-resistance signal recognition, transmission, and HR response. This suggests that the PTI defense system was activated at the transcriptome level after inoculation but was inhibited at the protein level in susceptible rice varieties.

## Conclusions

The study focused on the protein expression profiles of rice compatible and incompatible interactions at 24 hpi by *M*. *oryzae*. Our study reveals that the pathogen-associated molecular pattern (PAMP)-triggered immunity defense system may be activated at the transcriptome level but was inhibited at the protein level in susceptible rice varieties after inoculation. The results may facilitate future studies of the molecular mechanisms of rice blast resistance. Additionally, the differential expression profiles of the proteome and transcriptome may provide a new understanding of the interaction between rice and *M*. *oryzae*.

## Supporting information

S1 Fig Functional characterization of differentially expressed proteins(DEPs) in GY8 and LTH after inoculated with *M. oryzae*. The results are summarized in three main categories: biological process, molecular function, and cellular component by GO analysis. The bar on the left is the numbers of proteins in different categories and the bar on the right is the percents of protein number in different categories.
(TIF)

S2 Fig The gene expression pattern in GY8 and LTH after *M. oryzae* infection validated by quantitative RT-PCR. OsActin was used as the internal control, and the 2^–ΔΔC^T method was used to calculate relative expression levels. The red bar represents GY8 and the blue bar represents LTH. The gene name (ID) is below the picture.
(TIF)

S1 Table The replicates of every experiment. (XLSX)

S2 Table The list of differentially expressed proteins induced by *M. oryzae* invasion in GY8. (XLSX)

S3 Table The list of differentially expressed proteins induced by *M. oryzae* invasion in LTH. (XLSX)

S4 Table Antibodies used in western blotting for the validation of differentially expressed proteins. (XLSX)

S5 Table Primers used in qRT-PCR for the validation of differentially expressed genes. (XLSX)

S6 Table The differentially expressed proteins (DEPs) involved in specific metabolic processes in GY8 and LTH following *M. oryzae* inoculation. (XLSX)

S7 Table The list of differentially expressed genes induced by *M. oryzae* invasion in GY8 and LTH. (XLSX)

S8 Table The DEGs involved in specific metabolic processes in GY8 and LTH following *M. oryzae* inoculation identified by RNA-Seq. (XLSX)

S9 Table A list of TF genes that showed differential expression in GY8 and LTH after infection of *M*. *oryzae* identified by RNA-Seq. (XLSX)

S10 Table A list of RK genes that showed differential expression in GY8 and LTH after infection of *M*. *oryzae* identified by RNA-Seq. The IDs of the RK genes, log2 FC, and family of the genes are presented in the table. The IDs and gene family were retrieved from the rice kinase database.
(XLSX)

S11 Table A list of PR genes that showed differential expression in GY8 and LTH after infection of *M*. *oryzae* identified by RNA-Seq. The IDs of the TF genes, log_2_ FC, and family of the genes are presented in the table. The gene IDs and family names of the PR were obtained according to Dou 2014.
(XLSX)

S1 Raw Images (PDF)
