# Supplementary figures and images for: iTRAQ proteomics reveals the regulatory response to Magnaporthe oryzae in durable resistant vs. susceptible rice genotypes
Source: PLoS One. 2020 Jan 10;15(1):e0227470. doi: 10.1371/journal.pone.0227470 (PMC6954073; doi:10.1371/journal.pone.0227470)

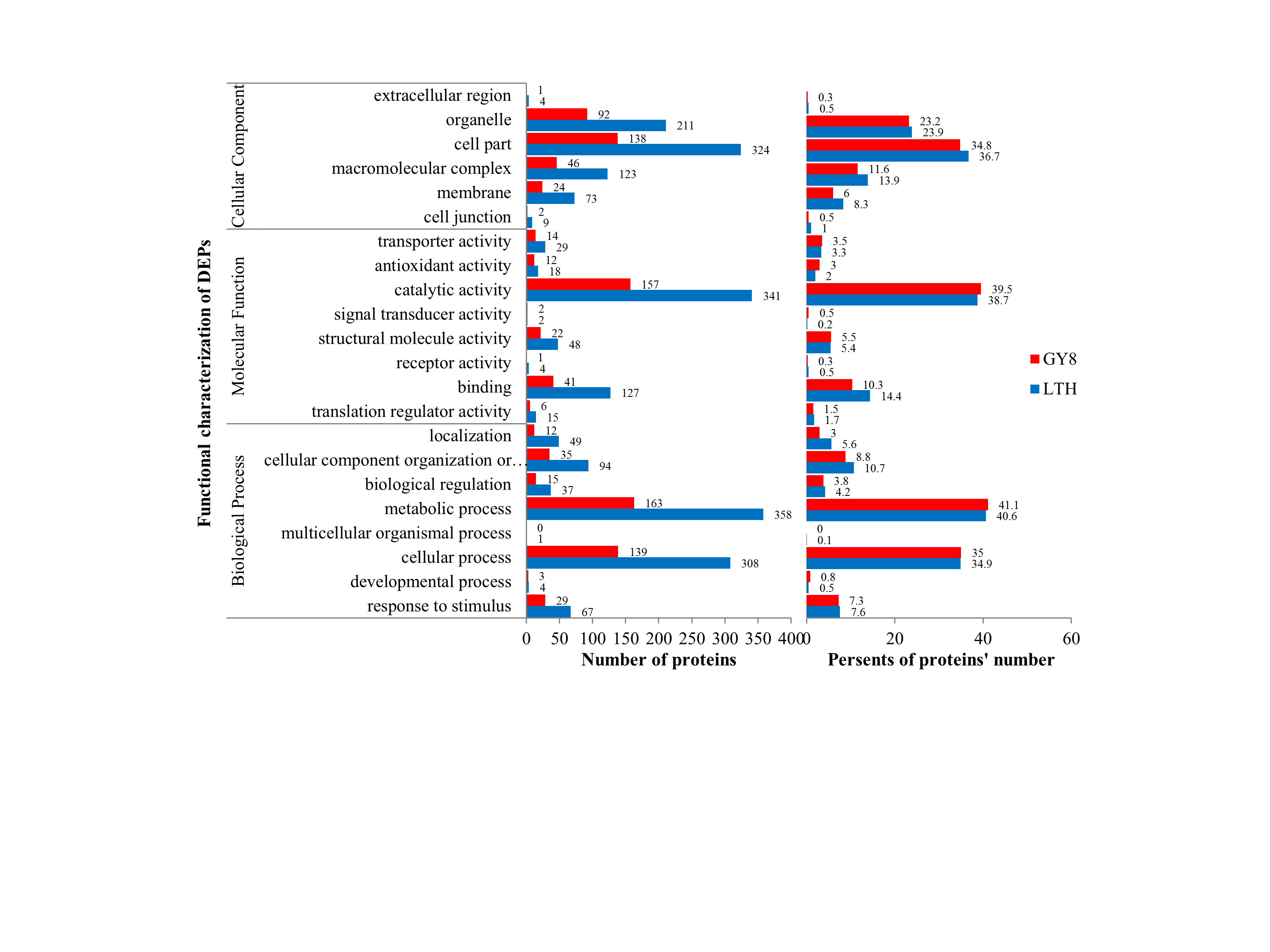

Supplement: S1 Fig — The results are summarized in three main categories: biological process, molecular function, and cellular component by GO analysis. The bar on the left is the numbers of proteins in different categories and the bar on the right is the percents of protein number in different categories. (TIF) [file pone.0227470.s001.tif]

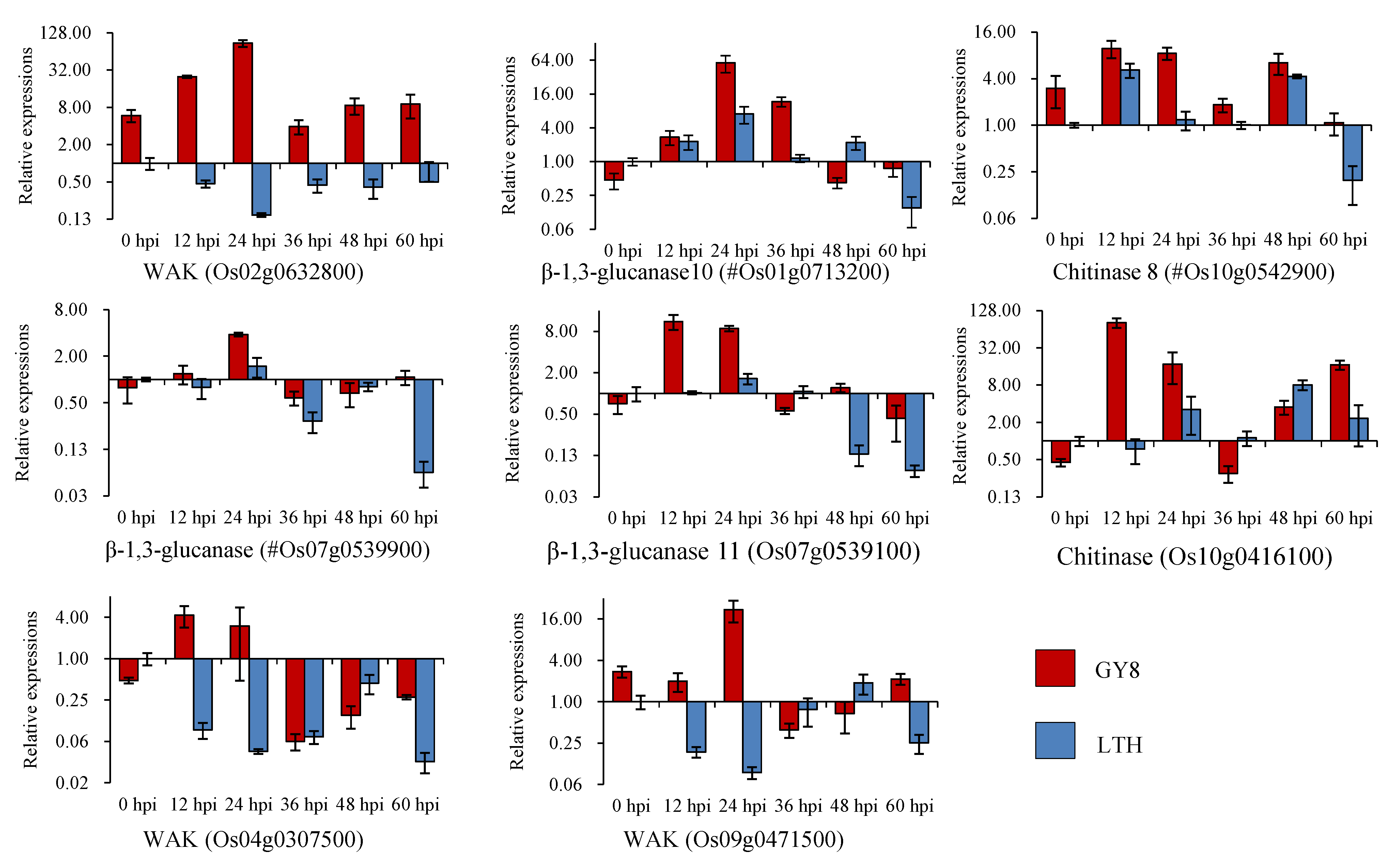

Supplement: S2 Fig — OsActin was used as the internal control, and the 2–ΔΔCT method was used to calculate relative expression levels. The red bar represents GY8 and the blue bar represents LTH. The gene name (ID) is below the picture. (TIF) [file pone.0227470.s002.tif]
