# Supplementary material for: iTRAQ proteomics reveals the regulatory response to Magnaporthe oryzae in durable resistant vs. susceptible rice genotypes
Source: PLoS One. 2020 Jan 10;15(1):e0227470. doi: 10.1371/journal.pone.0227470 (PMC6954073; doi:10.1371/journal.pone.0227470)

The original images for Fig. 5

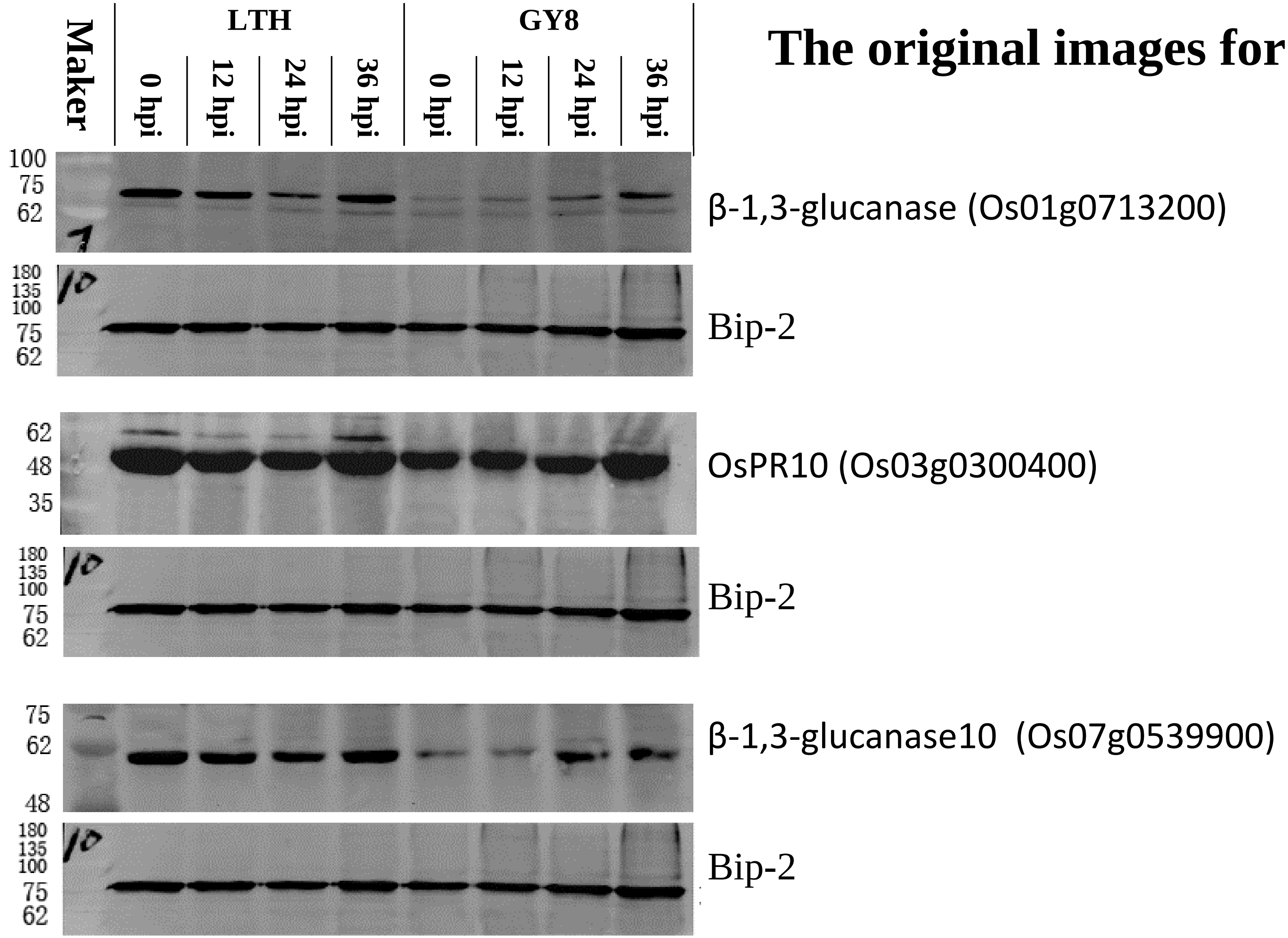

The original images for Fig. 5

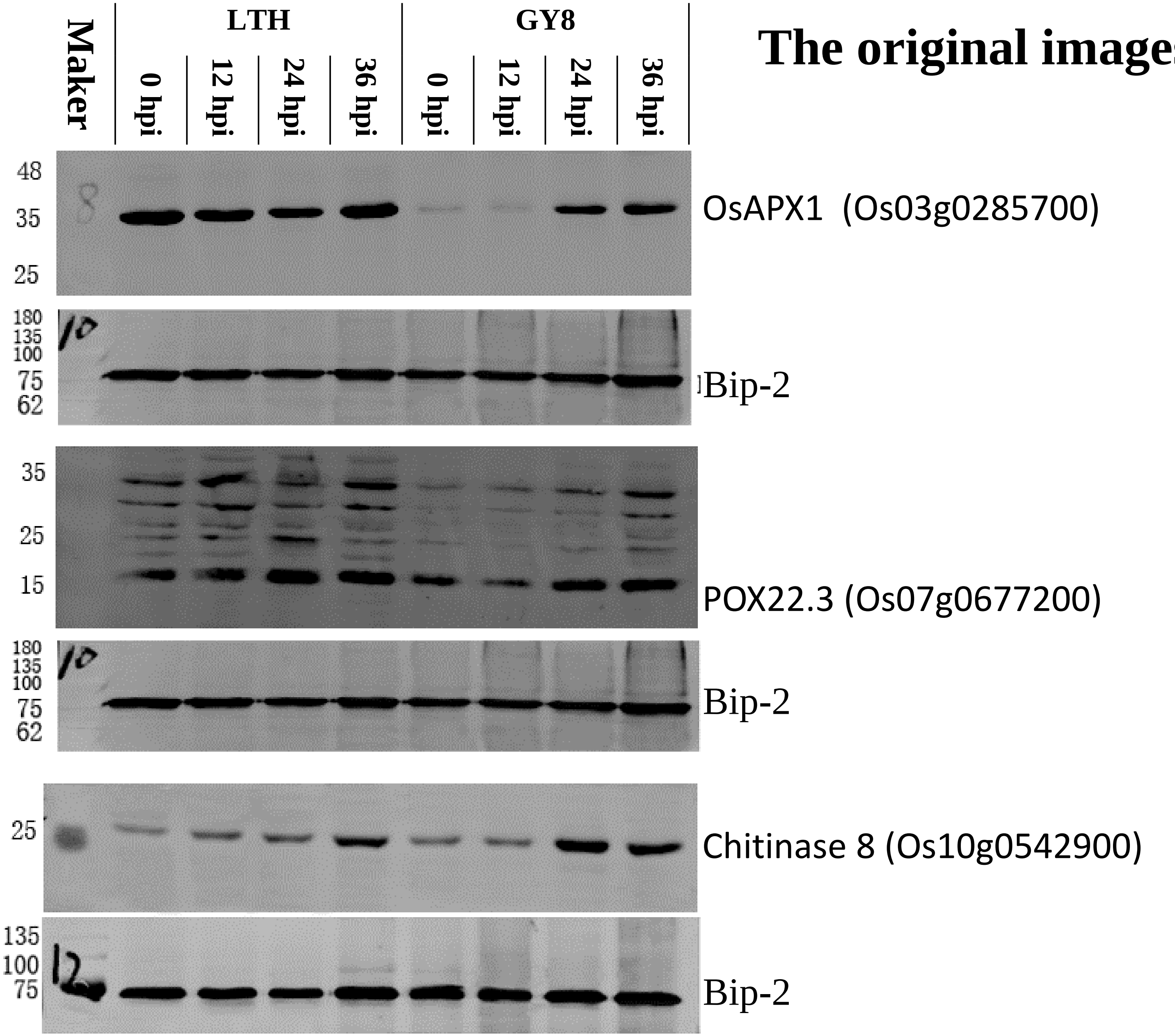

Supplement: S1 Raw Images — (PDF) [file pone.0227470.s014.pdf]
